# Whole-genome reference of *Dirofilaria immitis* from Australia to determine single nucleotide polymorphisms associated with macrocyclic lactone resistance in the USA

**DOI:** 10.1016/j.crpvbd.2021.100007

**Published:** 2021-01-13

**Authors:** Daisy Ching-Wai Lau, Stephanie McLeod, Sara Collaery, Selina Peou, Andy Truc Tran, Michelle Liang, Jan Šlapeta

**Affiliations:** Sydney School of Veterinary Science, Faculty of Science, The University of Sydney, NSW, 2006, Australia

**Keywords:** *Dirofilaria immitis*, Canine heartworm, Sydney, Resistance, SNP, Genomics

## Abstract

For the past 30 years, chemoprophylaxis with macrocyclic lactone (ML) anthelmintics has been the primary strategy for canine heartworm (*Dirofilaria immitis*) control in both the USA and Australia. ML-resistant *D. immitis* isolates have been confirmed to exist in the USA and studies have shown that 42 single nucleotide polymorphisms (SNPs) are associated with phenotypic ML-resistance. Currently, ML-resistance has not been reported in any Australian clinical cases of canine heartworm. The aim of the study is to determine whether the 42 SNPs associated with resistance to MLs in the isolates from the USA are present in adult heartworms from a clinical case in Australia. Five adult *D. immitis* obtained from a dog at post-mortem (Sydney, Australia) were sequenced using the Illumina sequencing technology. The genomic analyses revealed 6 out of the 42 SNPs associated with ML-resistance to be present in our samples, 3 out of the 6 SNPs identified were nonsynonymous SNPs but not in candidate genes for ML-resistance. ML-susceptibility profile was mixed using the 42-SNP and 10-SNP models, but the 5-SNP, 3-SNP and 2-SNP models demonstrated ML susceptibility for all five individuals. In this study, the first whole-genome reference of *D. immitis* from Australia establishes a new baseline for comparative studies and will be valuable for tracking ML-resistance emergence.

## Highlights

• *Dirofilaria immitis* from Sydney, Australia, sequenced using the Illumina NGS technology.
• New baseline for comparative studies for tracking ML-resistance emergence in Australia.
• Mixed ML-susceptibility profile using the 42-SNP and 10-SNP models.
• 5-SNP, 3-SNP and 2-SNP models demonstrated ML susceptibility.

## Graphical abstract

**Figure:**
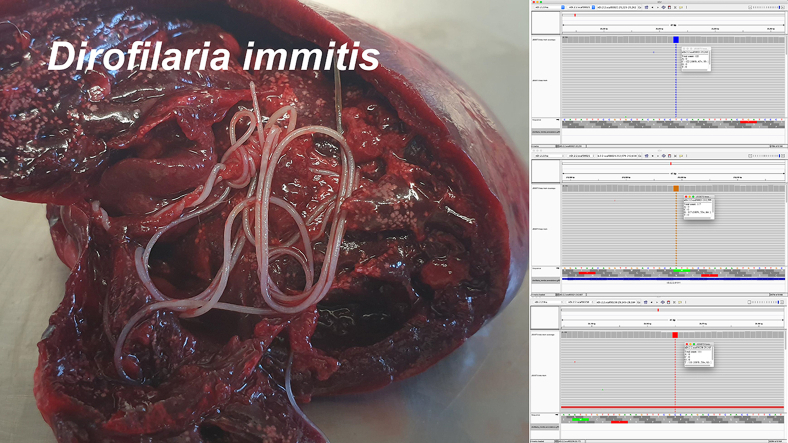

## 1 Introduction

*Dirofilaria immitis* (canine heartworm) are mosquito-borne filarial nematodes capable of causing cardiopulmonary disease. Canine heartworm disease affects dogs in Australia each year, particularly in Queensland (Nguyen et al., 2016). Chemoprophylaxis with macrocyclic lactone (ML) anthelmintics is the primary strategy for heartworm prevention with preventative products approved to be 100% efficacious (Hampshire, 2005). Within 20 years after the introduction of ML preventative anthelmintics, loss of efficacy (LOE) reports from the Mississippi Delta region in the USA have led to the speculation that *D. immitis* has developed ML-resistance (Atkins et al., 2014). Initially, distinguishing whether LOE cases are truly due to ML-resistance has proven challenging due to frequent owner non-compliance (Bowman, 2012). As the number of LOE cases continued rising, controlled efficacy studies were conducted to investigate ML-resistance of *D. immitis* and clinical isolates have been confirmed to be ML-resistant (Pulaski et al., 2014).

Genome comparison of ML-resistant isolates show they are distinct signatures from the ML-susceptible populations of *D. immitis*. Single nucleotide polymorphisms (SNPs) associated with failure to reduce microfilariae following ML prophylaxis have been identified (Bourguinat et al., 2011c, 2015). The initial genome comparison between susceptible and LOE *D. immitis* populations identified 186 SNPs that segregated with ML-resistance (Bourguinat et al., 2015). However, some of the 186 SNPs could be random mutations unrelated to ML-resistance (Bourguinat et al., 2015). Instead, by assessing the allele frequencies between well characterised ML-susceptible and ML-resistant isolates obtained from controlled efficacy studies, a subset of 42 ML-resistant markers was identified (Bourguinat et al., 2015). These appeared to better differentiate the ML-susceptible phenotype from the LOE and ML-resistant phenotype (Bourguinat et al., 2015).

ML anthelmintics were introduced into Australia in the 1990s and have been used in the USA and Australia for over 30 years (Hampshire, 2005; Orr et al., 2020). Research published from the USA demonstrated that ML-resistance is emerging; however, it is unknown whether the SNPs associated with ML-resistance in the isolates from the USA are present in Australian canine heartworms. The objective of this study is to determine whether any of the 42 SNPs associated with ML-resistance in the isolates from the USA are present in *D. immitis* adults from a clinical case in Australia.

## 2 Materials and methods

### 2.1 Sample processing and DNA extraction

Samples tested include five adult *D. immitis* JS5873 to JS5877 (P6/20 A-C: female and D-E: male) obtained at necropsy from a deceased dog donated to the Sydney School of Veterinary Science, The University of Sydney, in September 2020, as part of the Sydney School of Veterinary Sciences Animal Donation Programme. The adult dog came from Sydney, New South Wales, Australia, but the history of travel and rigorous treatment with ML products is unknown. Individually extracted nematodes from the heart were washed 3 times in phosphate-buffered saline (pH = 7.4) and 1 cm from between the first and second third of the anterior portion of the body was excised using a sterile scalpel blade for DNA isolation. The genomic DNA from samples JS5873 to JS5876 (*n* = 5) was extracted with a Monarch® Genomic DNA Purification Kit (New England Biolabs, Australia) as per the manufacturer's protocol.

### 2.2 Whole-genome sequencing

Extracted *D. immitis* DNA samples (A-C: 4.72–90.15 ng/μl and D-E: 0.27–1.06 ng/μl) were sent to Novogene (HK) Co., Ltd for indexing, library construction and whole-genome sequencing; JS5873 to JS5875 (A-D) were sequences at an expected depth of 10G while JS5876 and JS5877 were sequenced at a depth of 1G. Sequencing was performed with the HiSeq platform from Illumina® with PE150 paired-end reads.

### 2.3 Genome analysis and identification of SNP markers

Based on previous work, the 42 SNPs that appeared to better differentiate the ML-susceptible phenotype from the LOE and ML-resistance phenotype were selected for analysis (Bourguinat et al., 2015). The list of 42 SNPs evaluated in this study are available at LabArchives: https://doi.org/10.25833/8egx-j055. From the 42 SNPs, a subset of SNP loci (10-SNP, 5-SNP, 3-SNP and 2-SNP model) which appears to be promising markers for predicting ML resistance are used to predict the phenotypic response of JS5873 to JS5877 to ML treatment (Ballesteros et al., 2018; Bourguinat et al., 2017).

All bioinformatic tools used to identify DNA polymorphic sites at the 42 SNP loci in JS5873 to JS5877 adult *D. immitis* samples are available in the bioinformatics webserver, Galaxy Australia (usegalaxy.org.au) (Jalili et al., 2020). Quality of all raw paired-end sequencing reads (fastq) were checked with the FastQC tool (www.bioinformatics.babraham.ac.uk/projects/fastqc/). Raw sequencing reads were trimmed to have a Phred quality score (Q score) of 30 and filtered to have a minimum read length of 150 bp using Trimmomatic (Bolger et al., 2014). The filtered paired-end read sets were mapped to the nDi.2.2. *D. immitis* reference genome (www.nematodes.org/genomes/dirofilaria_immitis/) (Godel et al., 2012) using the Map with BWA-MEM tool and a Binary Alignment Map File (.bam) was created (Li & Durbin, 2009). Bam files were sorted based on scaffold alignment coordinates using the “SortSam” tool and the alignment statistics from the sorted BAM datasets were obtained using flagstat from Samtools (http://samtools.sourceforge.net/). Pileup files were created from the sorted BAM datasets using the “Generate pileup” tool and the consensus base and coverage at each of the 42 SNPs were extracted. FreeBayes, a haplotype-based variant detector was used to determine the total number of variants between the genomes of JS5873 to JS5877 and the nDi.2.2. *D. immitis* reference genome (Garrison & Marth, 2012).

### 2.4 Genome analysis of *Wolbachia* endosymbiont of *D. immitis* and *cox*1 mitochondrial DNA

For the genome analysis of the *Wolbachia* endosymbiont of *D. immitis*, the filtered paired-end read sets were mapped to the wDi.2.2 reference genome using the pipeline described above (www.nematodes.org/genomes/dirofilaria_immitis/) (Godel et al., 2012). FreeBayes was used on the sorted BAM datasets to determine the total number of variants between the genomes of JS5873 to JS5877 and the wDi.2.2 *Wolbachia* reference sequence.

The mitochondrial DNA (mtDNA) for each nematode was assembled from fastq data using the MITObim tool (https://github.com/chrishah/MITObim) (Hahn et al., 2013) as previously described (Červená et al., 2019), with the sequence of *D. immitis* complete mtDNA (NC_005305) as bait (Hu et al., 2003). The cytochrome *c* oxidase subunit 1 gene (*cox*1) was compared to *cox*1 haplotypes from Belanger and Perkins (2010). mtDNA genomes from mDi_Athens_2.1 and mDi_Pavia_2.1 were also included (www.nematodes.org/genomes/dirofilaria_immitis/) (Godel et al., 2012).

### 2.5 Nonsynonymous SNP data analysis

Gene annotations in the GFF3 (Generic Feature Format 3) file available at (https://parasite.wormbase.org/Dirofilaria_immitis_prjeb1797/Info/Index/) were used to annotate the nDi.2.2. *D. immitis* reference genome and visualised in IGV (Integrative Genomics Viewer) to assess whether the SNP was synonymous or nonsynonymous. The type of SNP, synonymous or nonsynonymous was determined by assessing whether the SNP altered the amino acid sequence of the annotated protein.

## 3 Results

Five *D. immitis* were individually sequenced and 90.21%–97.91% of the reads from the JS5873 to JS5877 samples could be aligned to the reference genome of *D. immitis* nDi.2.2. (88 Mb) (Table 1). Average coverage was 101–126× for JS5873, JS5874 and JS5875, compared with 11–12× for JS5876 and JS5877. Across all JS5873 to JS5877 *D. immitis* samples, total number of variations across the alignment against the nDi.2.2. *D. immitis* reference genome varied from 178,977 to 207,665 (Table 1).

**Table 1:** Summary of whole genome sequencing and mapping of *Dirofilaria immitis* from Australia

Sample ID	No. of reads	Alignment against nDi.2.2. *D. immits* [No. of reads (% of total reads)]	Average coverage (average no. of reads aligning at the specific position of the 42 SNPs)	Variants (total no. of variants across alignment against nDi.2.2. *D. immitis*)
JS5873	71,971,763	68,875,509 (95.70)	115	207,665
JS5874	73,578,378	72,039,266 (97.91)	126	206,681
JS5875	67,774,096	65,465,532 (96.59)	101	201,864
JS5876	8,704,246	8,418,566 (96.72)	11	176,559
JS5877	8,778,535	7,919,458 (90.21)	12	178,977

From the list of 42 SNPs evaluated in this study, 6 of the proposed SNPs which appear to better differentiate the ML-susceptible phenotype from the LOE and ML-resistance *D. immitis* samples were found in JS5873 to JS5877 samples (Table 2). All 5 samples had the same SNP at each of the 6 markers, apart from JS5875 to JS5877 at nDi.2.2.scaf00589_15334 and JS5875 to JS5876 at nDi.2.2.scaf00597_12915 where a heterozygous SNP is present. For the remaining 36 SNP markers, JS5873 to JS5877 samples are homozygous reference to the nDi.2.2. *D. immitis* reference genome. The nDi.2.2.scaf00597_12915 ML-resistance marker is part of the 10-SNP model. All other ML-resistance markers found in JS5873 to JS5877 samples are not part of the 5-SNP, 3-SNP or 2-SNP model.

**Table 2:** Distinct SNPs found within 42 SNPs *Dirofilaria immitis* reference

SNP Position	Reference Allele	JS5873	JS5874	JS5875	JS5876	JS5877
nDi.2.2.scaf00021_25243	T	C	C	C	C	C
nDi.2.2.scaf00021_212599	A	G	G	G	G	G
nDi.2.2.scaf00238_29165	C	T	T	T	T	T
nDi.2.2.scaf00589_15334	A	G	G	R^a^ (48:125)	R^a^ (7:9)	R^a^ (8:8)
nDi.2.2.scaf00597_12915	A	G	G	R^a^ (60:58)	R^a^ (13:10)	G
nDi.2.2.scaf01422_4176	G	A	A	A	A	A

a R stands for purine (adenine or guanine), proportion of A:G given in parentheses.

Based on the 42-SNP and 10-SNP models, the ML phenotypic response of JS5873 to JS5877 samples was considered mixed since both ML-resistance and ML-susceptible SNP markers are present (Table 3). For the 5-SNP, 3-SNP and 2-SNP model, the nucleotides at all SNP loci were homozygous reference to the nDi.2.2. *D. immitis* reference genome and the ML phenotypic response of JS5873 to JS5877 samples would be predicted to be ML-susceptible (Table 3).

**Table 3:** Predictive *Dirofilaria immitis* SNP models predicting ML resistance

Predictive model	JS5873	JS5874	JS5875	JS5876	JS5877
42 SNP	Mixed	Mixed	Mixed	Mixed	Mixed
10 SNP	Mixed	Mixed	Mixed	Mixed	Mixed
5 SNP	Susceptible	Susceptible	Susceptible	Susceptible	Susceptible
3 SNP	Susceptible	Susceptible	Susceptible	Susceptible	Susceptible
2 SNP	Susceptible	Susceptible	Susceptible	Susceptible	Susceptible

Three out of the six SNPs identified from JS5873 to JS5877 *D. immitis* samples were nonsynonymous SNPs (Table 4). SNPs at position nDi.2.2.scaf00021_212599, nDi.2.2.scaf00597_12915 and nDi.2.2.scaf01422_4176 lead to changes in the amino acid sequence and the predicted proteins altered were the GSK3B-interacting protein, K homology domain and protein kinase domain respectively. The SNP located at nDi.2.2.scaf00021_25243 was a synonymous change. The remaining two SNPs led to nonsynonymous changes, but the predicted protein has not been annotated.

**Table 4:** Synonymous SNPs and nonsynonymous SNPs at the 6 ML resistance marker sites for *Dirofilaria immitis*

SNP position	Amino acid	Amino acid change	Protein annotation
nDi.2.2.scaf00021_25243	CCT Pro	CCC Pro	Unannotated
nDi.2.2.scaf00021_212599	ATG Met	GTG Val	GSK3B-interacting protein
nDi.2.2.scaf00238_29165	CCA Pro	TCA Ser	Unannotated
nDi.2.2.scaf00589_15334	AAT Asn	AGT Ser	Unannotated
nDi.2.2.scaf00597_12915	ATT Ile	GTT Val	K homology domain
nDi.2.2.scaf01422_4176	GAT Asp	AAT Asn	Protein kinase domain

The genomic data of the *Wolbachia* endosymbiont of *D. immitis* (wDi) was included in 50,985 to 3,725,184 sequence reads mapped to the reference wDi genome (Table 5). From the mappling alignment, 4,924 to 7,962 SNPs were found between the JS5873 to JS5877 samples and the wDi.2.2 *Wolbachia* reference genome.

**Table 5:** *Wolbachia* endosymbiont of *Dirofilaria immitis* from Australia

Sample ID	No. of reads aligned against *Wolbachia* endosymbiont of *D. immitis* wDi.2.2	Variants (total no. of variants across alignment against wDi.2.2)
JS5873	3,725,184	7,407
JS5874	1,597,092	7,962
JS5875	2,034,077	7,570
JS5876	50,985	7,751
JS5877	1,016,571	4,924

To confirm the identity of *D. immitis*, the complete *cox*1 mtDNA was assembled. The *cox*1 mtDNA from all five *D. immitis* samples from Sydney, Australia was the same. There were 56, 11 and 19 differences across the alignment (13,820 positions) with the reference *D. immitis* mtDNA (NC_005305) from Australia (Hu et al., 2003), mDi_Athens_2.1 from Athens, Georgia, USA, and mDi_Pavia_2.1 from Pavia, Lombardy, Italy, respectively. The *cox*1 of the *Dirofilaria immits* from Sydney was 100% identical with mDi_Athens_2.1 and mDi_Pavia_2.1 as well as haplotypes AL59 and WI04 (USA) and MX03 (Mexico).

## 4 Discussion

This study utilised a whole-genome sequencing approach to characterise the ML-susceptibility profiles of *D. immitis* in Sydney, Australia, based on the genotyping data available from the USA. For nearly three decades, ML anthelmintics have been used as the primary strategy for canine heartworm prevention in the USA and Australia but ML-resistant isolates have only been recovered in the USA (Bourguinat et al., 2015; Orr et al., 2020; Pulaski et al., 2014). With no studies published from Australia, there is a gap in scientific knowledge regarding any potential LOE or ML-resistant cases in the Australian canine heartworm population. In studies from the USA, phenotypic characterisation of clinically infected dogs and controlled efficacy studies utilising animal models enabled them to establish SNPs as predictors of ML-resistance (Bourguinat et al., 2015; Pulaski et al., 2014). With the recent re-emergence of *D. immitis* in Queensland, genetic variants previously found associated with ML-resistance in the isolates from the USA may also be present in clinical cases of canine heartworm in Australia (Nguyen et al., 2016). In this study, the first whole-genome reference sequences of *D. immitis* from Australia have been sequenced using modern NGS techniques. These reference sequences will become a valuable tool to refine the genetic identity of ML-resistance in comparative studies.

In recent years, NGS technologies have been used for high-throughput whole-genome sequencing for genetic marker discovery (Davey et al., 2011). Read coverage is a crucial parameter to evaluate variant calling accuracy and comparison of whole-genome sequencing data showed that SNPs achieved > 95% of concordance at 17.6× coverage (Kishikawa et al., 2019). Following this standard, the accuracy of the 6 SNPs found in JS5873 to JS5875 *D. immitis* samples is > 95%. In this study, ∼16 million sequencing reads were required to achieve a coverage of 17.6× . This number of sequencing reads should be the minimum level of data acquired in future studies to obtain accurate genomic data for subsequent analyses of SNPs associated with ML-resistance.

In a previous study, a whole-genome sequencing approach between ML-susceptible, LOE and ML-resistant clinical isolates of *D. immitis* from the USA identified a set of 42 SNPs to differentiate between the different ML-susceptibility profiles (Bourguinat et al., 2015). Using a similar approach, 6 out of the 42 ML-resistance SNP markers were found in JS5873 to JS5877 *D. immitis* samples obtained from a clinical case of canine heartworm from Australia. These adult canine heartworm samples did not show a similar genetic profile to previous phenotypically well-characterised *D. immitis* ML-susceptible and ML-resistant isolates, which suggests that the 42 SNPs may be geographically specific and therefore cannot be used globally (Bourguinat et al., 2015, 2017). In *Caenorhabditis elegans*, a SNP is expected to appear by chance in the nuclear genome at an average rate of 2.7 × 10^−9^ per-generation (Denver et al., 2009). If this mutation rate is representative of random base substitutions in *D. immitis*, then ∼96,000 mutations is expected to occur by chance across the genome (Denver et al., 2009). In our study, 178,977–207,665 genetic variants were found between the JS5873 to JS5877 *D. immitis* samples and the nDi.2.2. *D. immitis* reference genome. Using the mutation rate of *C. elegans*, ∼46% of the genetic variants seen across the genomes are not due to chance. This level of non-random genetic variability could be due to population structure (geographical separation, genetic bottleneck, selective sweep, or selection by ML anthelmintics), which could affect the viability of the SNP markers previously identified to be promising in detecting ML-resistant cases (Gilleard & Beech, 2007).

The set of 42 SNPs found to be associated with phenotypic ML resistance have not been identified as the actual cause of ML resistance (Bourguinat et al., 2015). Instead, the 42 SNPs may be surrogate markers for ML resistance. This is further supported by our results where 3 out of the 6 SNPs led to nonsynonymous changes in protein-coding regions of the genome. Nonsynonymous changes occurred in the GSK3B-interacting protein, K homology domain and the protein kinase domain. But none of these proteins are known to be candidate genes of ML-resistance (Bourguinat et al., 2011a, 2011b; Wolstenholme et al., 2016).

Our inability to evaluate the ML phenotypic response of JS5873 to JS5877 samples with the set of 42 SNPs prompted further evaluation of the ML-susceptibility profile with use of the 10-SNP, 5-SNP, 3-SNP and 2-SNP model (Ballesteros et al., 2018; Bourguinat et al., 2017). Recent studies have assessed the allele frequencies between well characterised ML-susceptible and ML-resistant isolates obtained from controlled efficacy studies and different combinations of SNP markers more highly correlated with the ML-resistant phenotype have been identified (Ballesteros et al., 2018; Bourguinat et al., 2017). The study by Bourguinat et al. (2015), showed that a number of genetic markers in the set of 42 SNPs, such as nDi.2.2.scaf00021_212599 would fail to clearly distinguish between ML-susceptibility profiles. Use of the 42 SNPs as genetic markers to test for ML susceptibility was not reliable as single nucleotide substitutions at nDi.2.2.scaf00021_212599 and various other SNP loci were seen in all susceptible, LOE and resistant phenotypic groups (Bourguinat et al., 2015, 2017). Interestingly, the 10-SNP model, previously found to be highly correlated to ML-resistance in the isolates from the USA, failed to determine the ML-phenotypic response of our samples (Bourguinat et al., 2017). However, the 5-SNP, 3-SNP and 2-SNP models predicted the ML-susceptibility profile of the Australian *D. immitis* samples.

The adult canine heartworm samples utilised in this study have not been subjected to any ML-phenotypic testing. Thus, our results do not provide unequivocal evidence that other *D. immitis* samples with the same genetic profile are susceptible to MLs. To eliminate this issue, future studies would benefit from obtaining both phenotype and genotype of microfilariae from infected dogs. The ML phenotypic response of microfilariae can be predicted using the 7-day microfilariae suppression test (Geary et al., 2011). Isolation of ML-susceptible and suspected ML-resistant microfilariae followed by whole-genome sequencing and analysis will be necessary for phenotypic and genotypic characterisation of canine heartworms from Australia.

The filarial nematode *D. immitis* harbours bacterial (*Wolbachia*) endosymbionts that could become resistant to the antibiotic doxycycline - an established component for treatment of heartworm-infected dogs (Belanger & Perkins, 2010; Shin et al., 2020). The whole genome sequencing approach is suitable for evaluating the genomic sequence of both the nematode as well as its endosymbiont. The *Wolbachia*-endosymbiont has been shown to include 67 loci specifically found in resistant *D. immitis* isolates but not in susceptible *D. immitis* USA isolates (Shin et al., 2020). Around 3,000 SNPs in the wDi sequences have been found between drug-sensitive and resistant isolates of *D. immitis* (Shin et al., 2020). Further studies including additional *D. immitis* sampling and genome sequencing are needed to understand why there is almost twice the number of SNPs in Australian wDi.

## 5 Conclusion

This is the first study to analyse whole-genome reference sequences of adult *D. immitis* originating from Australia. While finding evidence of ML-resistance in *D. immitis* is still pending, whole-genome sequencing and bioinformatics provide an affordable and scalable approach for future studies. Efforts to design reliable and sensitive *in vitro* tests for resistance remains unsuccessful (Evans et al., 2013; Maclean et al., 2017). Therefore, identifying reliable genetic markers to predict ML response may be the only way to monitor the extent and spread of ML-resistant isolates in epidemiological studies. Much remains unknown about the distribution of ML-resistant heartworm populations in Australia and the extent to which the efficacy of ML anthelmintics is threatened. This creates a need for further research into whether ML-resistance is present in the canine heartworm population in Australia. Improvements to the approach used in this study combined with the 7-day suppression test will be essential to refine ML-resistant genetic profiles using Australian strains of *D. immitis*.

## Funding

This research was supported by the 10.13039/100007456Canine Research Foundation and Dogs Victoria, Australia.

## CRediT author statement

Daisy Ching-Wai Lau: Methodology, Validation, Formal analysis, Data curation, Writing - Original Draft, Writing - Review & Editing. Sara Collaery: Methodology, Formal analysis, Writing - Original Draft. Michelle Liang: Methodology, Formal analysis, Writing - Original Draft. Stephanie Mcleod: Methodology, Formal analysis, Writing - Original Draft. Selina Peou: Methodology, Formal analysis, Writing - Original Draft. Andy Tran: Methodology, Formal analysis, Writing - Original Draft. Jan Šlapeta: Conceptualization, Validation, Investigation, Resources, Data Curation, Writing - Review & Editing, Supervision, Funding acquisition. All authors read and approved the final manuscript.

## Data availability

Raw fastq sequence data was deposited at SRA NCBI BioProject PRJNA681066. SNP data tables and additional data are available at LabArchives: https://doi.org/10.25833/8egx-j055.

## Declaration of competing interests

The authors declare that they have no known competing financial interests or personal relationships that could have appeared to influence the work reported in this paper.
